# *Rosa x hybrida*: A New Tool for Functional Food Development with Triple-Negative Breast Antitumoral Implications

**DOI:** 10.3390/ijms27020907

**Published:** 2026-01-16

**Authors:** Lorenzo Rivas-Garcia, Tamara Y. Forbes-Hernández, Pablo Cristóbal-Cueto, David Tébar-García, Alfonso Salinas-Castillo, Ana Cristina Abreu, Ignacio Fernández, Pilar Aranda, Juan Llopis, Elena Nebot-Valenzuela, Eva M. Galan-Moya, Cristina Sánchez-González

**Affiliations:** 1 Department of Physiology, Institute of Nutrition and Food Technology “José Mataix Verdú”, Biomedical Research Centre, University of Granada, 18016 Armilla, Spainparanda@ugr.es (P.A.); jllopis@ugr.es (J.L.); 2 Instituto de Biomedicina (IB_UCLM), Universidad de Castilla-La Mancha, Campus de Albacete, 02008 Albacete, Spain; 3 Instituto de Investigación Biosanitaria ibs.GRANADA, 18012 Granada, Spain; 4 Grupo Mixto de Oncología Traslacional UCLM-GAI Albacete, Universidad de Castilla-La Mancha-Servicio de Salud de Castilla-La Mancha, Campus de Albacete, 02008 Albacete, Spain; 5 Department of Analytical Chemistry, Campus Fuentenueva, Faculty of Sciencies, University of Granada, 18071 Granada, Spain; 6 Department of Chemistry and Physics, Research Centre CIAIMBITAL, University of Almería, 04120 Almería, Spain; acabreu@ual.es (A.C.A.); ifernan@ual.es (I.F.); 7 Department of Physiology, School of Medicine, Complutense University of Madrid, 28040 Madrid, Spain; 8 Facultad de Enfermería, Universidad de Castilla-La Mancha (UCLM), Campus de Albacete, 02006 Albacete, Spain

**Keywords:** ROS, cancer, NMR, apoptosis, autophagy, polyphenols

## Abstract

Edible flowers have garnered increasing attention due to their high content of bioactive compounds, making them promising candidates for biomedical and functional food applications. This work evaluated the metabolomic data of fresh *Rosa x hybrida* petals, revealing seven types of metabolites, including amino acids, organic acids, vitamins, sugars, phenolic acids, and flavonoids. Notably, quercetin, kaempferol and their derivatives were the main flavonoids determined. Furthermore, in vitro studies were conducted to evaluate the potential antiproliferative effects against triple-negative breast cancer (TNBC). Thus, the methanolic extract derived from *Rosa x hybrida* petals demonstrated significant antitumoral activity against both sensitive and resistant TNBC cells, as evidenced by reduced MTT metabolization, colony formation, and wound healing activity. Furthermore, the cell death mechanism associated with the petal extract was studied. The antiproliferative activity was mediated by reactive oxygen species generation, triggering cell death mechanisms such as apoptosis and autophagy. In conclusion, these results propose *Rosa x hybrida* could be a new tool for nutraceuticals and functional food production.

## 1. Introduction

Over the past decade, there have been substantial pieces of evidence of the intricate connection between human nutrition and health. This burgeoning awareness has piqued the interest of consumers, who are increasingly interested in the benefits offered by a well-balanced diet in reducing the incidence of several non-transmissible diseases such as cardiovascular diseases, diabetes, and specific types of cancer [1]. Consequently, the development of novel ingredients and foods, with applications in the production of nutraceuticals and functional food, has become a paramount pursuit in the field of food technology.

In this context, products derived from the matrices of fruits and vegetables have been lauded for their potential to promote health and reduce the incidence of chronic and degenerative diseases [2]. A growing field of interest in the food and pharmaceutical industries is the integration of edible flowers into functional food design [3]. These blooms are gaining attention due to their potential implications in mitigating chronic diseases. Extracts derived from *Rosa x hybrida* and *Tulbhagia violacea* have demonstrated anti-proliferative effects against tumor cell lines, achieved through autophagy induction and the generation of reactive oxygen species (ROS). Additionally, these extracts have shown promise in combating Alzheimer’s Disease (AD), with their efficacy attributed to their rich phenolic compound content, primarily featuring quercetin and its derivatives [4,5].

Polyphenols constitute a diverse and numerous group of chemical structures (more than 8000 individual phenolic compounds), stemming primarily from shikimic acid, and are integral to plant metabolism, influencing both plant growth and defense mechanisms against diseases and pathogens [6]. While their traditional role in medicine has been linked to their antioxidant properties, their potential as therapeutic agents has expanded to include anti-tumor and antibiotic activities [6].

Edible flowers, including various species of *Rosa*, are increasingly recognized for their potential health-promoting properties, which can be attributed to their rich content of bioactive compounds such as flavonoids and polyphenols. Several species of *Rosa* sp. have been employed as therapeutic agents motivated by their chemical composition. For example, *Rosa roxburghii* is employed in traditional Chinese medicine, especially in the province of Guizhou [7]. The primary rationale for its utilization in biomedical research lies in its polyphenolic content. Various types of roses, including *Rosa x hybrida* [4], *Rosa roxburghii* [7,8], *Rosa canina* [9], *Rosa sterilis* [10], and *Rosa centifolia* [11], have been characterized, including petals [4], leaves [12] and roseships (fruits) [13]. Different species and cultivars of *Rosa* sp. have demonstrated in vitro antiproliferative effects in various cancer cell lines [14]. *Rosa damascena* extracts reduced the viability of cervical carcinoma cells in a concentration- and time-dependent manner [15], while *Rosa x hybrida* petal extract induced autophagy and apoptosis, leading to inhibition of ovarian cancer cell proliferation [4]. Similarly, extracts from *Rosa rugosa* petals showed strong antiproliferative activity against cervical carcinoma and breast carcinoma cells [12]. These findings underscore the importance of further in vitro studies on different rose varieties to elucidate the mechanisms by which rose metabolites, such as flavonoids and polyphenols, exert anticancer effects.

Nevertheless, the market for edible flowers remains an emerging niche, with its production primarily concentrated in Asian countries. Restructuring these crops to cater to the production of functional foods holds the promise of facilitating their integration into Southern European countries, including Spain, Portugal, and Italy, where well-established agricultural and food industries can support the diversification of crops and the enhancement of product value.

This work aims to assess the potential anti-tumoral effects of a methanolic extract obtained from *Rosa x hybrida*, with the ultimate goal of developing novel nutraceuticals and functional foods. Furthermore, the antiproliferative activity of petal extracts has been studied in triple-negative breast cancer cells (TNBC). TNBC, a type of breast cancer that lacks expression of progesterone and estrogen receptors and the human epidermal growth factor receptor (HER2), is associated with the poorest prognosis in cancer. This unique challenge arises from the lack of specific drug targets [16]. In this context, natural products and other techniques such as image analysis [17], and new drug strategies [18] have emerged as promising therapeutic tools for addressing the complexity of TNBC.

## 2. Results and Discussion

### 2.1. Rose Petal Characterization

**Figure 1:**
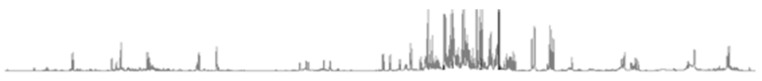
^1^H NMR (600 MHz) spectrum of the petals of *Rosa x hybrida*.

Table 1 and Figure 1 provide an initial overview of the metabolite assignments obtained for *Rosa x hybrida*. The reported data include chemical shift values, signal multiplicities, and coupling constants corresponding to each identified metabolite or metabolite class. Metabolite identification was achieved through the interpretation of one- and two-dimensional homo- and heteronuclear Nuclear Magnetic Resonance (NMR) spectra, including ^1^H-NOESY, ^1^H–^1^H TOCSY, ^1^H–^1^H COSY, ^1^H–^13^C edited HSQC, and ^1^H–^13^C HMBC experiments. These analyses were complemented by reference to the Chenomx NMR Suite and publicly accessible spectral databases such as COLMAR and HMDB, as well as previously published literature.

Up to 37 distinct compounds have been identified in the fresh petals of *Rosa x hybrida*. They have been categorized into seven different groups based on biochemical classification. These categories include amino acids, sugars, organic acids, vitamins, phenolic compounds, flavonoids, and others. Among these, flavonoids are the category with the highest number of individual compounds reported, with up to 10 individual compounds identified via NMR analysis in the rose petals. Notably, the predominant flavonoids present in these edible petals are kaempferol and quercetin. Furthermore, it is worth highlighting that up to nine different amino acids have been identified. However, for other categories, fewer individual compounds have been reported, with only one vitamin (ascorbic acid) and two individual phenolic compounds identified (Gallic acid and Syringic acid).

*Rosa* sp. is formed by around 200 species that grow naturally or are cultivated [19]. Some of these species are relevant in horticulture in Europe, Asia, North America, and the Middle East [19,20]. Several species of *Rosa* sp. have been used in health applications motivated by their chemical composition. For example, *Rosa roxburghii, Rosa x hybrida* [4], *Rosa roxburghii* [7,8], *Rosa canina* [9], *Rosa sterilis* [10], and *Rosa centifolia*. In all cases, these species have been reported to exhibit a similar polyphenolic profile. It has been observed that the primary components are flavonoids, such as quercetin, gallocatechin, epigallocatechin, and kaempferol. Additionally, flavanoles like epicatechin and catechin, as well as terpenes such as roseoside, and phenolic acids like ellagic acid, ethyl gallate and caffeic acid, have been identified [4,8]. Our findings partially align with prior literature. Thus, fresh petals of *Rosa x hybrida* included in this work exhibited flavonoids like quercetin, kaempferol, epicatechin, and catechin, along with phenolic acids such as gallic acid and syringic acid However, others polyphenols did not find. This discrepancy may be attributed to the detection technique used. NMR techniques applied for food characterization have gained interest due to the possibility of performing a cost-effective approximation of fingerprinting studies and their high reproducibility compared to other metabolomic approaches [21]. However, other studies utilized UPLC/MS and LC/MS, and they identified a greater number of individual polyphenols and concluding that these techniques are more efficient than NMR. Furthermore, our research group assessed the chemical composition of a methanolic extract obtained from these petals, reporting a chemical composition similar to that obtained from fresh petals. These results confirm that the extract of *Rosa x hybrida* maintains a similar composition of phenolic compounds as the fresh petals.

It is noteworthy that the extraction method influences the number of phenolic compounds in the extract. Methanolic or phenolic extraction methods yield approximately twice the number of individual polyphenols as aqueous methods [4,8,22]. This phenomenon is consistent with other flower species, such as *Tagetes erecta* [23]. Recently, it has been reported that the use of technologies like Microwave Hydrodiffusion and Gravity Extraction can enhance the extraction of polyphenols from *Rosa canina* compared to conventional chemical extraction methods [24].

Furthermore, the findings obtained in the present study suggested that other components related to the antioxidant activity such as ascorbic acid constituted the structure of the rose petals.

Subsequently, a methanolic extract was synthesized from the edible petals of *Rosa x hybrida*. The production of extract facilitates the inclusion of plant components in foods. These extracts provide high stability, improve their physicochemical parameters, simplify the manipulation and make possible their introduction in some steps of the functional food production process [25]; actually, extract from *Rosa roxburghii* has been included in the formulation of an apple juice revealing several anti-browning activity [26], improving the juice’s quality and the acceptance of consumers.

In addition to NMR-based profiling, the extract obtained from *Rosa x hybrida* petals was previously analyzed using Ultra Performance Liquid Chromatography coupled to Tandem Mass Spectrometry (UPLC MS/MS) [4] to complement and validate the metabolite assignments. Comparison of the two datasets revealed that four metabolites—catechins, kaempferol glycosides, quercetin glycosides, and gallic acid—were detected by both NMR and UPLC. These types of metabolites are generally stable throughout plant extraction procedures [27].

### 2.2. Antitumoral Screening Assays

The biological assays were designed to evaluate the functional relevance of the metabolites identified in Table 1 and Figure 1. In particular, the high content of flavonoids and phenolic acids in the methanolic extract provides a biochemical rationale for the antiproliferative effects observed against TNBC cells.

**Figure 2:**
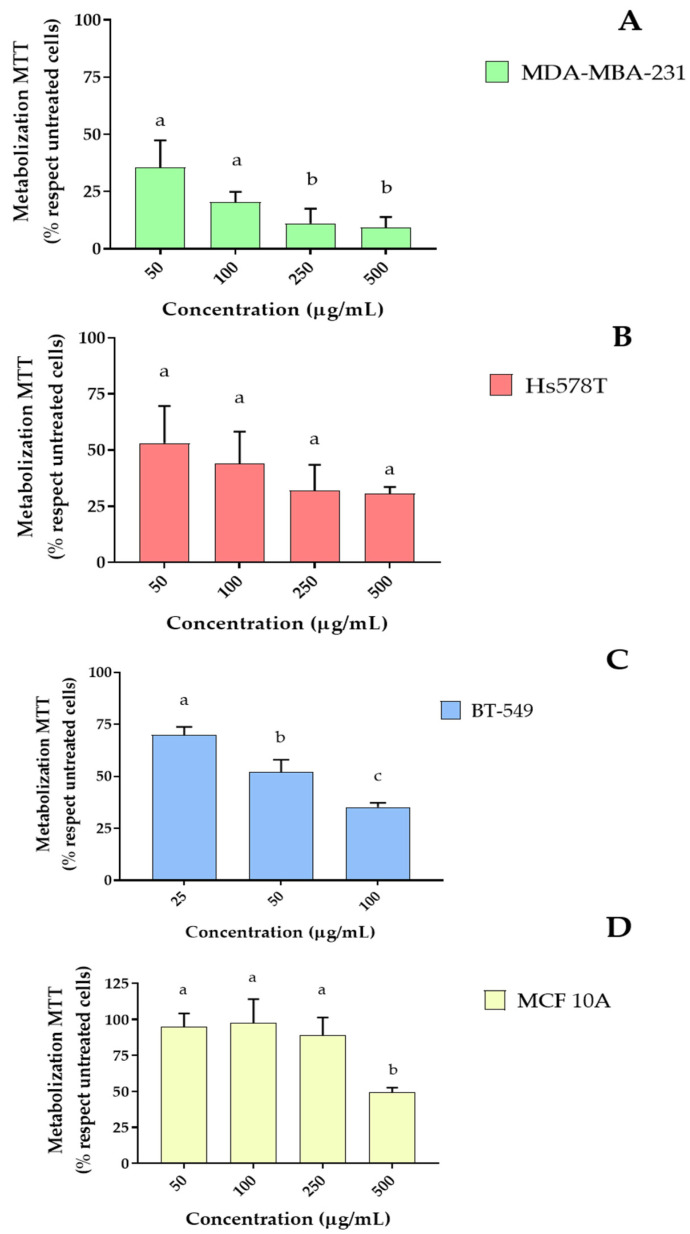
MTT assays after 72 h exposition of methanolic *Rosa x hybrida* extract. (**A**) MDA-MBA-231 cell line; (**B**) Hs578T cell line; (**C**) BT-549 cell line; (**D**) MCF10A cell line. and MCF 10A. Mean ± SD. Different letters mean statistically significant differences between groups. *p* < 0.05.

Thus, to evaluate the potential biomedical applications of the *Rosa x hybrida* extract, screening test were carried out to assess its antitumoral activity against various TNBC cell lines, both sensitive (MDA-MB-231 and BT-549) and resistant to cisplatin (Hs578T). Firstly, MTT was carried out. Figure 2 shows the effect of *Rosa x hybrida* extract in MTT metabolization after 72h of incubation. For all the conditions, the extract produced a dose-dependent decrease in its metabolism. Notably, MDA-MB-231 was the most sensitive cell line; for example, the dose of 50 µg/mL petal extract reduced MTT metabolism by approximately 70% in MDA-MB-231 cells, and this dose decreased the metabolism by only 50% in the other lines. Subsequently, the IC_50_ parameter for each cell line were determined. MDA-MB-231 reported IC_50_ of 23.5 µg/mL, the IC_50_ for BT-549 was 66.7 µg/mL; finally, Hs578T cells had a score of 61.7 µg/mL.

**Figure 3:**
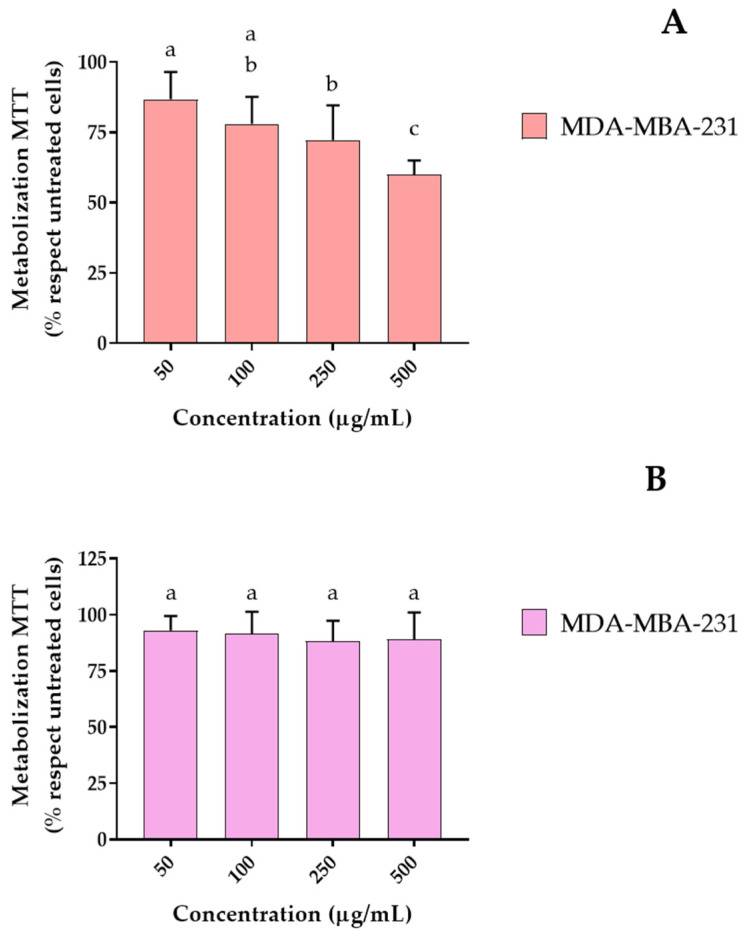
MTT assays in MDA-MB-231 cells after 48 h (**A**) and 24 h (**B**) exposure to methanolic *Rosa x hybrida* extract. Mean ± SD. Different letters indicate statistically significant differences between groups (*p* < 0.05).

To address the time-dependent characteristics of the extract, additional MTT assays were performed on the most sensitive cell line, MDA-MB-231, at 24 h and 48 h of exposure (Figure 3). A clear temporal trend was observed: the antiproliferative effect increased progressively with longer incubation times. While the reduction in metabolic activity was moderate at 24 h and more pronounced at 48 h, the maximum inhibitory effect was reached at 72 h, confirming that the effect of the extract is both dose- and time-dependent.

Previous studies evaluated the antiproliferative capacity of some rose extracts. Thus, *Rosa x hybrida* and *Rosa damascena*, had proved their efficiency as antiproliferative agents against cervical carcinoma cells and ovarian tumoral cells [4,15,28]. Moreover, *Rosa canina* extract reported antitumoral activity for lung and prostate cells [29]. However, these authors described IC_50_ values higher than those obtained in the present study for TNBC. Up to date, little evidence described the antitumoral effect to edible rose flowers against TNBC. It is noteworthy that the extract exhibited its highest efficacy in MDA-MB-231 cells following 72 h of treatment. Therefore, this cell line was selected for subsequent experiments.

Additionally, the rose extract was incubated with a model of non-malignant breast epithelial cells (MCF10A) to assess its toxicity. This extract did not alter the MTT parameters compared to untreated cells (Figure 2D). The absence of toxicity of these rose extracts has been previously reported by other authors, using both cellular models and *C. elegans models*. In these cases, treatment of the worms with extracts obtained from petals of *Rosa x hybrida*, did not modify lifespan parameters [4]. Furthermore, the lack of toxicity for other extracts synthesized from *Rose* sp. petals had been proved previously. For example, the extraction of free and bound phenolic fractions from *Rosa roxburghii* Tratt pomace reported an absence of toxicity and antioxidant activity in worm models [8].

Supplemental Figure S1 shows optical microscopy images of MDA-MB-231 cells treated with the petals extract, revealing a notable alteration in cell morphology. Treated cells exhibited an elongated and filariform structure and significantly decreased the cell number. Previous studies on flower extracts, such as Al-Oqair and collaborator reported no significant changes in HCF7 cells after 24 h exposition with a methanolic extract of *Rosa damascene*. However, in our study with a longer exposure time (72 h), pronounced alterations in cell morphology were reported. For further understanding, cells were stained by toluidine blue (Supplemental Figure S2). This staining technique did not report differences between the control group and cells exposed to the extract, but in this group, a small number of cells exhibited an engrossed aberrant structure (detailed Supplemental Figure S2).

**Figure 4:**
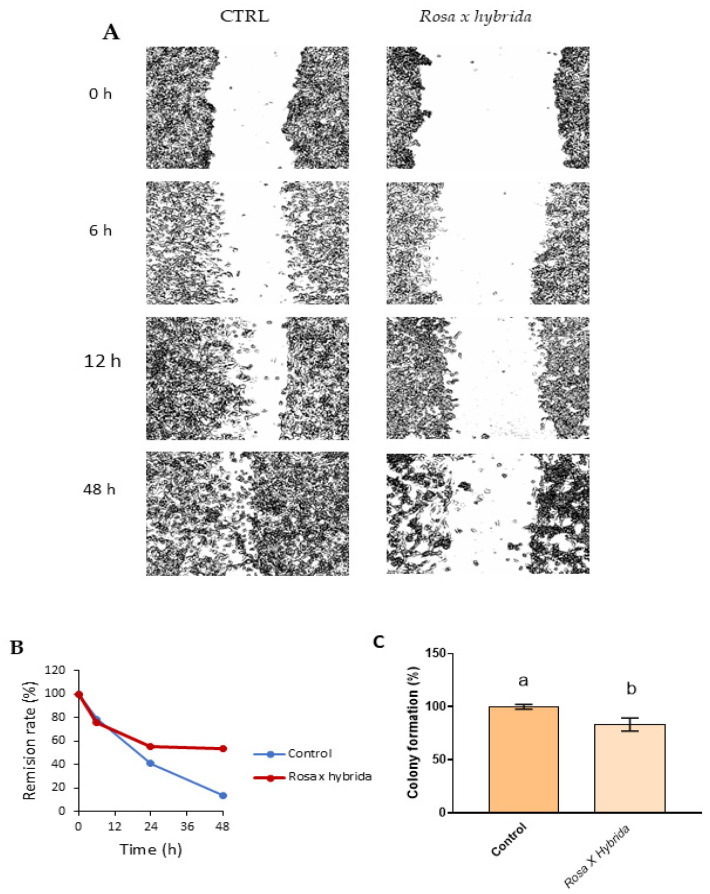
(**A**) Representative images from wound healing assay of MDA-MBA-231 cell culture treated with IC_50_ petals extract and untreated cells. (**B**) Summary graph showing typical wound healing rate. (**C**) colony formation of tumoral cells MDA-MBA-231. Mean ± SD. Different letters mean statistically significant differences between groups. Control: cells treated with the solvent (Milli-Q water). *p* < 0.05.

Furthermore, the screening antitumoral activity of the extract included the wound healing rate and clonogenic assay test against MDA-MB-231 cells. TNBC represents approximately 15% of all tumor cases detected and is characterized by the worst breast cancer prognosis, a high tumor rate and metastatic activity. Figure 4A shows the characteristic pictures of tumor cells after scratch; under the conditions studied, the incubation of cells with petal extract decreased the re-epithelization rate compared to untreated cells, i.e., after 48 h incubation, the remission rate of TNBC cells treated with the extract was 60%, and untreated cells remitted approximately 100% (Figure 4B). This fact could be associated with the content of flavonols; in that way, some dietary flavonoids, such as kaempferol or apigenin, could inhibit the metastatic activity of TNBC cells in a dose-dependent manner [30]. Moreover, the extract reduced the formation of new colonies (Figure 4C). Hence, developing new functional foods supplemented with edible flower extract could be a new tool for managing oncologic patients [31].

The observed dose- and time-dependent inhibition of TNBC cell proliferation, reduced colony formation, and impaired migration can be attributed to the metabolites identified in the *Rosa x hybrida* extract. These metabolites are known to modulate apoptosis, ROS production, and cytoskeletal organization, providing a direct mechanistic link between the metabolomic profile (Table 1, Figure 1) and the antitumoral.

A limitation of this study is that the molecular mechanisms underlying the observed inhibition of cell migration were not directly investigated. Specifically, analyses such as Western blot detection of migration-related proteins (e.g., MMP-9, E-cadherin) or Transwell migration assays were not performed. Therefore, the results provide phenotypic evidence of antimigratory activity, but do not elucidate the specific molecular pathways involved.

### 2.3. Cell Death Mechanism

Previous literature studies that describe the antiproliferative activity of extracts obtained from Rosaceae usually did not describe the cell death mechanism involved. The present work aims to report the action of edible rose flowers against TNBC. Describing the cell death mechanism not only is a key point to facilitate its translation into the management of oncologic patients through its inclusion in diet nutraceuticals and/or functional foods, but also could contribute to developing a new economic niche for functional food production based on these edible flower petals.

**Figure 5:**
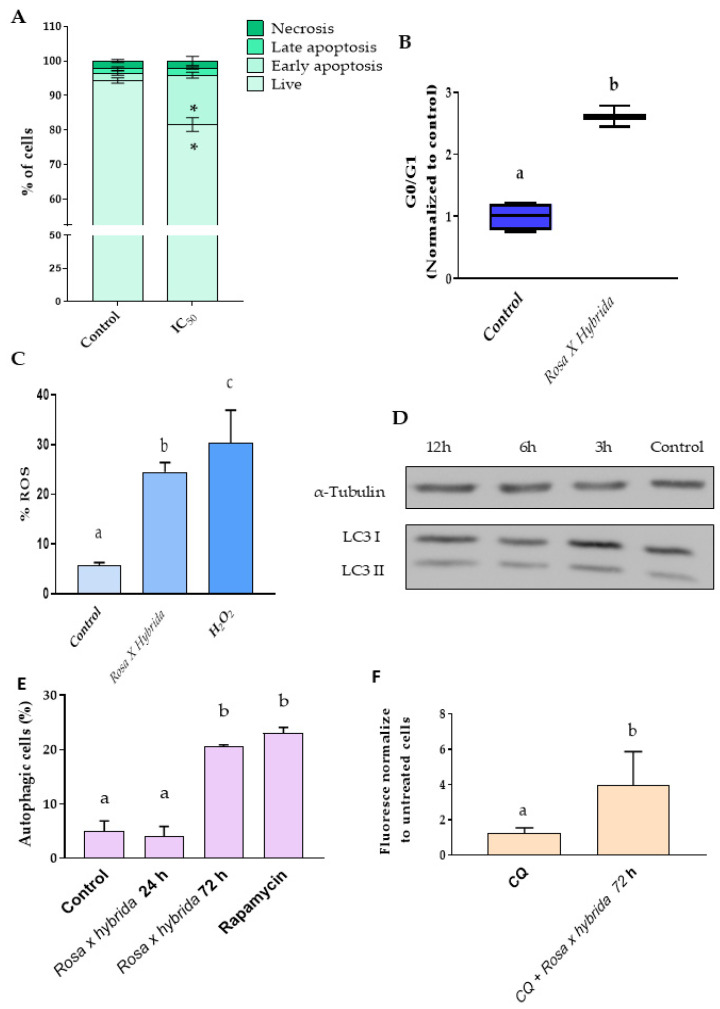
(**A**) Evaluation of apoptosis/necrosis by flow cytometry. * vs. untreated cells; (**B**) MDA-MBA-231 cells arrested in G0/G1. Different letters mean statistically significant differences between groups. *p* < 0.05; (**C**) ROS production measured by flow cytometry. H_2_O_2_ 25 μM (positive control); (**D**) Induction of autophagy evaluated by flow cytometry. Rapamycin (500 nM) was used as positive; (**E**) LC3II/LC3I and α-tubulin protein representative picture. α-tubulin was employed as the protein control (**F**) Autophagy induction determined by fluorescence using a microplate reader in after the treatment with 50 μM of chloroquine (CQ) for 12 h. Different letters mean statistically significant differences between groups. The intensity was normalized to untreated cells. Control: cells treated with the solvent (Milli-Q water) * *p* < 0.05.

The first approach to understand the role of *Rosa x hybrida* extract was to measure the levels of apoptosis/necrosis by cell staining employing Annexin V and propidium iodide (IP) by flow cytometry. Figure 5A shows a statistically significant increase in early apoptotic cells between untreated cells (control) and cells exposed to petal extract for 72 h and no differences were reported between the other groups. In addition, the induction of apoptosis was confirmed by measuring G0/G1 arrest; thus, the treatment of cells with petal extract significantly increased this parameter (Figure 5B), and the arrest of this cell phase is intended to be a consequence of apoptosis [32]. These results agreed with previous literature that reflected apoptosis as the main cell death mechanism associated with an extract obtained from *Rosa canina* in colonic tumoral cells. These authors reported a decrease in hTERT gene expression, evidencing the role of telomerases in apoptosis regulation [29].

On the other hand, the results obtained under the present conditions evidenced an increase in reactive oxygen species (ROS) produced by 72 h of petal extract exposure (Figure 5C). An elevation in ROS levels has been identified as a key trigger of apoptosis in cancer cells and is considered a potential strategy for cancer therapy [33,34]. The link between apoptosis and ROS mediated by a methanolic extract of *Rosa damascene* was previously described by Al-Oqail, M.M. and coworkers [28], who confirmed ROS proliferation by the increase in LPO and the decrease in GSH. The results obtained under the present conditions confirmed this hypothesis and suggest that this effect could be mediated by the flavonoids of the petals.

**Figure 6:**
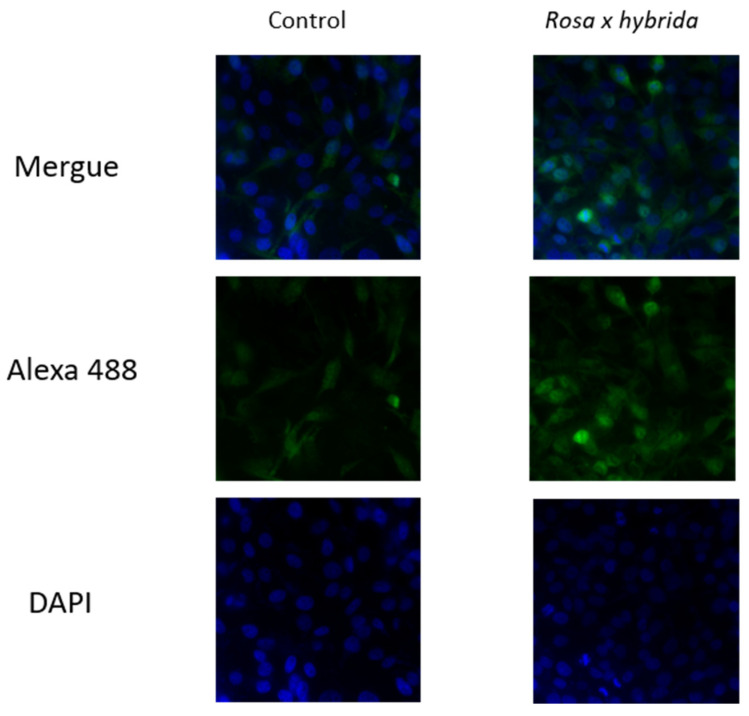
Fluorescence microscopy images of MDA-MBA-231 cells exposed to IC_50_ *Rosa x hybrida* extract. Immunofluorescence staining of cleaved caspase-3.

Additionally, immunofluorescence analysis revealed a significant increase in cleaved caspase-3 levels in MDA-MB-231 cells after 72 h of petal extract treatment (Figure 6), providing specific molecular confirmation of apoptosis. This finding corroborates the Annexin/PI staining and G0/G1 arrest data, strengthening the evidence that the extract induces apoptotic cell death in TNBC cells.

Notable, several studies have demonstrated that the main metabolites identified in *Rosa x hybrida* extract by NMR and UPLC MS/MS can directly induce apoptosis via caspase-dependent pathways in various cancer models. For instance, kaempferol has been reported to trigger apoptosis through the activation of caspase-3 and PARP cleavage in breast and colon cancer cells [35,36]. Quercetin similarly induces apoptosis in MDA-MB-231 and other TNBC models by upregulating cleaved caspase-3 and promoting G2/M or G0/G1 arrest [37]. Catechin has also been associated with increased caspase-3 activity in hepatocellular and colorectal carcinoma cells [38], while gallic acid has been shown to enhance cleaved caspase-3 and caspase-9 expression in human leukemia and lung cancer cells [39]. Moreover, this ability to activate the caspase-dependent pathway has also recently been reported in other edible flower species [40].

Nevertheless, the results obtained regarding the activation of apoptosis did not suggest that apoptosis was the only cell death mechanism implicated. Consequently, autophagy was evaluated as an alternative cell death mechanism; autophagy is a catabolic process that enables the recycling of cell components. Relations between autophagy and cancer are controversial; thus, some authors suggest this process as a way to eliminate toxic elements and facilitate tumoral cell survival [41]. In contrast, autophagy could be associated with a cell death mechanism [42]. Autophagy can be modulated by stress signals, such as ROS induction [43]. Thus, the ROS generated in TNBC cells by exposure to the extract could induce autophagy. To assess this process, the LC3BII/LC3BI ratio was measured at shorter exposure times (i.e., 6 h); Figure 5D shows that the treatment with the *Rosa x hybrida* did not alter the rate of LC3BII/LC3BI. These findings differ from previous reports indicating that extracts from these petals can induce autophagy in ovarian tumor cells [4]. Subsequently, a flow cytometry assay demonstrated that incubation of cells with the rose extract for 72 h increased autophagy levels, similar to the positive control (Figure 5F). Additionally, treatment with the *Rosa x hybrida* petal extract tended to reverse the chloroquine-mediated inhibition of autophagy.

Together, these results suggest that autophagy induction by the rose petal extract occurs primarily during long-term incubation (72 h).

Although the LC3BII/LC3BI ratio did not show significant changes at early time points, and pre-treatment with chloroquine did not lead to notable differences upon extract exposure, the overall increase in autophagy detected by the kit suggests that *Rosa x hybrida* extract can induce autophagic activity. These results may indicate a modest or tightly regulated induction of autophagy rather than a complete autophagic flux blockage. The absence of significant changes in LC3BII/LC3BI could reflect the limitations of this single marker in capturing subtle or transient autophagic events. Therefore, while the extract appears to stimulate autophagic processes, the magnitude of the effect may require longer exposure times or additional molecular markers (such as p62 degradation or tandem mCherry-GFP-LC3 reporters) for more sensitive detection of autophagic flux integrity.

This study demonstrates that *Rosa x hybrida* petal extract exerts antitumoral effects in TNBC cells, including induction of apoptosis (cleaved caspase-3, Annexin/PI staining, G0/G1 arrest), increased ROS production, long-term autophagy activation, and inhibition of colony formation and migration. Nevertheless, several limitations should be acknowledged. Autophagic flux was assessed only partially, and subtle or transient effects may not have been fully captured. The wound healing assay suggests antimigratory activity, but molecular mechanisms were not fully explored. Importantly, all findings are derived from in vitro experiments, and no in vivo validation has been performed. In particular, studies in TNBC tumor-bearing animal models would be necessary to evaluate the systemic efficacy, pharmacokinetics, tissue distribution, and potential toxicity of the extract. Such experiments are critical to confirm whether the bioactive effects observed in vitro can translate into meaningful antitumoral activity and safety in a physiological context. Therefore, while these findings highlight the bioactive potential of the extract, further in vivo studies are essential before functional food applications can be fully substantiated.

## 3. Materials and Methods

### 3.1. Edible Flowers

Petals from *Rosa x hybrida* were harvested in the Innoflower fields located in Zaragoza, Spain. These petals were cultivated in Spain. Only petals that were uniform in appearance and free from damage, shriveling, or immaturity were selected, and all samples were collected on the same day. Upon arrival at the laboratory, they were immediately stored at −80 °C until further processing. Prior to extraction or analysis, the petals were freeze-dried under vacuum conditions using a Telstar lyophilizer (Madrid, Spain).

### 3.2. Nuclear Magnetic Resonance (NMR)

#### 3.2.1. Sample Preparation for NMR

A total of 20 mg of dried rose petals was extracted with 1 mL of a solvent mixture composed of methanol-d4 and KH_2_PO_4_ buffer in D_2_O (80:20, *v*/*v*, pH 6.0), containing 0.01% (*w*/*w*) 3-(trimethylsilyl)propionic-2,2,3,3-*d*_4_ acid (TSP) as a chemical shift reference and 90 µM sodium azide (NaN_3_) to inhibit enzymatic activity. The extraction procedure involved vortexing the samples at 600 rpm for 20 min, followed by centrifugation at 13,500 rpm for 5 min. Subsequently, 500 µL of the resulting supernatant was carefully transferred into pre-dried 5 mm NMR tubes for analysis.

#### 3.2.2. NMR Analysis

NMR spectra were acquired at 293 ± 0.1 K using a Bruker Avance III 600 spectrometer (Billerica, MA, USA), operating at a proton frequency of 600.13 MHz, equipped with a 5 mm QCI quadruple resonance pulse field gradient cryoprobe. Measurements were performed without sample rotation, applying 4 dummy scans followed by 32 acquisition scans. Acquisition parameters were set as: fid size = 64 K, spectral width = 20.5 ppm, acquisition time = 2.73 s, relaxation delay = 10 s, receiver gain = 57, FID resolution = 0.25 Hz, and mixing time = 10 ms. To suppress the residual water signal, a presaturation pulse sequence (Bruker 1D noesygppr1d) was applied, irradiating the H_2_O frequency during the recycle and mixing delay periods; this sequence is widely employed in metabolomics studies. Spectra were automatically phased, baseline-corrected, and referenced to the TSP signal at 0.0 ppm. The spectrometer transmitter was locked to the CH_3_OH-d_4_ frequency. Both acquisition and spectral processing were conducted using TOPSPIN software (version 3.1). Observed signal multiplicities were denoted as follows: s = singlet; d = doublet; dd = doublet of doublets; ddd = doublet of doublets; t = triplet; m = multiplet.

### 3.3. Petal Extract Production

For the synthesis of *Rosa x hybrida* extract, 500 mg of petals were combined with 1 mL of an extraction solution composed of methanol, Milli-Q water, and concentrated formic acid (80:20:0.1, *v*/*v*/*v*). The mixture was homogenized for 2 min at medium–high speed using an Ultraturrax T25 homogenizer (Janke & Kunkel, IKA Labortechnik, Staufen, Germany). Extraction efficiency was enhanced by stirring the suspension at 22× *g* with an ARE magnetic stirrer (VELP Scientifica, Usmate, Italy) for 2 h in the dark at room temperature. The resulting mixture was centrifuged twice at 2400× *g* for 15 min each to remove solid material. Supernatants were subsequently filtered through a 0.45-μm Minisart filter (PBI International, Milan, Italy) and collected in 5.0 mL amber glass vials. For downstream applications, fully concentrated and dried using a rotary evaporator to ensure complete removal of any residual methanol. The resulting dry extract was then stored in aliquots at –80 °C. Importantly, for all subsequent biological assays, this dried extract was resuspended exclusively in sterile Milli-Q water, which served as the vehicle administered to the cells.

### 3.4. Cell Culture Conditions

All cells were obtained from the American Type Culture Collection (ATCC, VA, USA). Triple-negative cancer cells MDA-MB-231 (ATCC HTB-26), BT-549 (ATCC HTB-122) and Hs578T (ATCC HTB-126) and healthy cells MCF10A (ATCC CRL-10317) were grown as adherent monolayers in Dulbecco’s Modified Eagle’s Medium (DMEM) containing 10% Foetal Bovine Serum (FBS), 100 U/mL penicillin, 100 μg/mL streptomycin and 2 mM L-glutamine for MDA-MB-231 and Hs578T. For maintaining the BT-549 cells, they were grown using the Roswell Park Memorial Institute Medium (RPMI 1640) supplemented with 10% Foetal Bovine Serum (FBS), 100 U/mL penicillin, 100 μg/mL streptomycin and 2 mM L-glutamine (Sigma Aldrich, Merck KGaA, Darmstadt, Germany). MCF10A cells were maintained in DMEM/F12 supplemented with 5% horse serum, 20 ng/mL EGF, 0.5 μg/mL hydrocortisone, 10 μg/mL insulin, 1% non-essential amino acids and antibiotics.

All reagents were purchased from Sigma Aldrich, Merck (Darmstadt, Germany).

### 3.5. Preliminary Antitumor Activity

#### 3.5.1. MTT Assay

For the MTT metabolization assay, 1 × 10^4^ cells were seeded in a 96-well plate for 24 h. Subsequently, the culture medium media were replaced and adjusted with the corresponding concentration of (summarized in Supplemental Table S1). After 72 h of incubation, formazan production by the metabolization of 3-(4,5-dimethylthiazol-2-yl)-2,5-diphenyltetrazolium bromide (MTT) was determined using spectrophotometry. Control cells were treated with Milli-Q water (vehicle control).

#### 3.5.2. Colony Formation

For the colony formation assay, 2 × 10^5^ cells were seeded in a 6multiwell plate. The following day, the cells were treated with the IC_50_ of petal extract. Then, the cells were harvested, quantified, and replated into 6-well plates at a density of 5 × 10^3^ cells per well. 7 days later, the cells were immobilized using 0.5% glutaraldehyde for 15 min, followed by staining with 0.05% crystal violet for 10 min. Colony formation was subsequently quantified using ImageJ software (v. 1.54p, Wayne Rasband National Health Institutes, Kensington, NY, USA).

#### 3.5.3. Wound Healing Assay

For the wound healing assay, MDA-MB-231 cells were grown to a complete cell monolayer, and a scratch was performed using a sterile 100 μL micropipette tip on the surface of the plates. Subsequently, the medium was replaced with DMEM without FBS and phenol red, and the cells were treated with the IC_50_ of rose extract or vehicle control (Milli-Q water) and incubated for 48 h at 37 °C and 5% of CO_2_. Cells were photographed at 0, 6 h, 12 h, and 48 h, and the remission percentage was calculated employing the appropriate macro for the wound healing assay of ImageJ software (Wayne Rasband National Health Institutes, Kensington, MD, USA).

#### 3.5.4. Optical Microscopy

MDA-MB-231 cells were exposed to the IC_50_ of the petal methanolic extract for 72 h. Then, the cells were fixed and stained with toluidine blue according to the procedure described by de Campos, B. and Mello, M.L. [44].

### 3.6. Cell Death Mechanism by Flow Cytometry

#### 3.6.1. Apoptosis/Necrosis

For the apoptosis/necrosis assay, MDA-MB-231 cells were cultured at a density of 1 × 10^5^ cells/mL in 6-multiwell plates. After 24 h, the medium was refreshed, and the cells were incubated for 72 h with the IC_50_ petals extract. Subsequently, the cells were collected, washed twice with cold PBS (Phosphate-Buffered Saline) and centrifuged at 120× *g* for 5 min. The cells were stained in the dark for 1 h at 4 °C with propidium iodide/RNase staining solution (Immunostep S.L., Salamanca, Spain). Flow cytometric analysis was conducted using a FACSCanto II cytometer (BD Biosciences, Milpitas, CA, USA), and the resulting data were processed with FACSDiva software (v 9.0).

#### 3.6.2. G0/G1 Arrest

To measure G0/G1 arrest, MDA-MB-231 cells were seeded in 6-multiwell plates at 1 × 10^5^ cells/mL. 24 h later, the culture medium was refreshed, and the cells were exposed to the IC_50_ *Rosa x hybrida* extract for 72 h. Then, cells were harvested, washed twice with cold PBS, and fixed in ice-cold 70% ethanol for 30 min. Fixed cells were then centrifuged at 120 × g for 5 min, resuspended in PBS containing 2% BSA, and incubated with a propidium iodide/RNase staining solution (Immunostep S.L., Salamanca, Spain) in the dark at 4 °C for 1 h. Cell cycle analysis was performed using a FACSCanto II flow cytometer (BD Biosciences, Franklin Lakes, NJ, USA), and the data analysis was performed using FACS Diva software (BD Biosciences, Franklin Lakes, NJ, USA).

#### 3.6.3. Autophagy

The induction of autophagy induction was determined by the CYTO-ID Autophagy detection kit 2.0 (Enzo Biochem Inc., Lausen, Switzerland) according to the manufacturer’s instructions. Briefly, 1 × 10^5^ MDA-MB-231 cells/mL were seeded in 6-well plates. Then, 24 h before, the cell medium was refreshed and supplemented with the IC_50_ rose extract, and the cells were incubated for 24 h and 72 h, respectively. Rapamycin 50 nm for 16 h was employed as the autophagy inducer for the positive control. Control cells were treated with Milli-Q water (vehicle control). Subsequently, cells were detached from the wells using trypsin and washed with 1× assay buffer. The cells were then incubated in DMEM containing Green Stain solution for 30 min at room temperature. After staining, cells were collected, washed again with 1× assay buffer, and resuspended in fresh 1× assay buffer prior to analysis in a FACSCanto II cytometer (BD Biosciences, Franklin Lakes, NJ, USA). The respective software FACSDiva (BD Biosciences, Franklin Lakes, NJ, USA) was used to analyze the results of the experiments.

For autophagy flux assessment, cells were treated with 50 µM chloroquine for 12 h. Then, cells were exposed to the extract of petals at its IC_50_ concentration for 72 h. Subsequently, autophagy induction was measured by fluorescence using a microplate reader ((Biotek, VT, USA), excitation 480 nm-emission 530 nm), and the CYTO-ID Autophagy Detection Kit 2.0, following the manufacturer’s instructions.

### 3.7. Western Blot

MDA-MB-231 cells were seeded at 5 × 10^5^ cells/100 mm dish and treated with IC_50_ petal extract for 72 h. After treatment, cells were washed three times with cold PBS, and proteins were extracted using a cold lysis buffer containing 20 mM Tris–HCl (pH 7.0), 140 mM NaCl, 50 mM EDTA, 10% glycerol, 1% Nonidet P-40, 1 μM pepstatin, 1 μg/mL aprotinin, 1 μg/mL leupeptin, 1 mM PMSF, 1 mM sodium orthovanadate, 25 mM β-glycerophosphate, and 50 mM NaF. Insoluble material was removed by centrifugation at 10,000× *g* for 10 min. Protein concentrations were determined using a commercial kit (Thermo Scientific, Waltham, MA, USA) according to the manufacturer’s instructions.

For Western blotting, 60 μg of protein was separated on 12% SDS–PAGE and transferred onto polyvinylidene difluoride membranes (Merck Millipore, Darmstadt, Germany). Membranes were blocked for 1 h in 1× Tris-buffered saline (TBS; 100 mM Tris, pH 7.5, 150 mM NaCl) containing 0.05% Tween 20 and 1% bovine serum albumin, then incubated overnight with primary antibodies: anti-LC3B (Santa Cruz, CA, USA), anti-γH2AX (Santa Cruz Biotechnology, Santa Cruz, CA, USA), and anti-tubulin (Santa Cruz, CA, USA).

Bound primary antibodies were detected using horseradish peroxidase-conjugated secondary antibodies (anti-rabbit or anti-mouse; Bio-Rad, Hercules, CA, USA, and GE Healthcare, Buckinghamshire, UK) diluted 1:1000 in 1× TBS with 0.05% Tween 20 and incubated for 30 min at room temperature. Protein bands were visualized using an ECL Plus Western Blotting Detection System (GE Healthcare, Buckinghamshire, UK).

### 3.8. Inmunofluorescence Microscopy

MDA-MB-231 cells cultured on glass coverslips were exposed to the petal extract for a 72 h period. After treatment, the cells underwent PBS washing and were subsequently preserved using paraformaldehyde at room temperature. Following fixation, the monolayers were washed again and placed in a blocking solution containing Triton X-100 and BSA. The samples then proceeded through several brief incubations before exposure to an antibody against cleaved caspase-3 (Cell Signaling Technology, Danvers, MA, USA; 1:200). Afterward, the coverslips were rinsed with PBS supplemented with BSA and incubated with an Alexa Fluor 488–conjugated secondary antibody (1:1000). Nuclear counterstaining with DAPI was carried out prior to mounting. Fluorescent images were acquired with a Leica DMIRE-2 inverted epifluorescence microscope (Wetzlar, Germany). The procedure was repeated in three independent experiments.

### 3.9. Statistical Assays

For in vitro studies, descriptive statistical parameters (means and standard deviations of experiment) were obtained for each of the variables studied. The means of independent variables were statistically compared among the groups. All analyses were performed using SPSS 26.0. (SPSS, Chicago, IL, USA). Each experiment was performed in triplicate. Differences were considered statistically significant at a probability level < 5%. GraphPad Prism 6 software was used to generate the graphics.

For NMR analysis, spectra were processed and bucketed using AMIX 3.9.12 (Bruker BioSpin GmbH, Rheinstetten, Germany). Buckets were generated by integrating 0.04 ppm intervals and subsequently normalized by scaling each peak’s intensity to the total intensity across the δH 0.2–10.0 ppm region, correcting for vertical scale variations due to differing water content. The δH 5.08–4.60 ppm region was excluded from the analysis to eliminate interference from the residual H2O signal. Multivariate data analysis was then conducted using SIMCA-P software (v. 15.0, Umetrics, Sweden), assuming normally distributed data.

## 4. Conclusions

The present results described that *Rosa x hybrida* fresh petals contained up to seven different types of metabolites determined by NRM spectroscopy and could be classified as amino acids, organic acids, vitamins, sugars, phenolic acids, flavonoids, and others. Especially relevant were the contents of quercetin and kaempferol and their derivatives due to their numerous potential applications in biomedical sciences.

Furthermore, a methanolic extract obtained from these petals showed antitumoral activity against resistant and sensitive triple-negative breast cancer cells, reduced the colony formation rate and modulated the remission rate determined by the wound healing assay, which are indicative of potential antitumoral applications. In addition, the extract increased ROS production in cells after 72 h of treatment, which could mediate the activation of apoptosis and autophagy as tumor cell mechanisms.

In summary, the results obtained guaranteed *Rosa x hybrida* as a new ingredient for functional food formulation or its inclusion in other industries, such as nutraceuticals or pharmacy.

## Figures and Tables

**Table 1:** Peak assignment of metabolites identified on *Rosa x hybrida* CD_3_OD: D_2_O KH_2_PO_4_ buffer (80:20, *v*/*v*) extracts by NMR.

Metabolite	Chemical Shifts (ppm), Multiplicity and Coupling Constants (Hz)
**Amino acids**	
Valine	1.06 (d, J = 7.1 Hz), 1.01 (d, J = 7.1 Hz)
Isoleucine	1.03 (d, J = 7.1 Hz), 0.96 (t, J = 7.3 Hz)
Threonine	1.33 (d, J = 6.6 Hz), 3.57, 4.18
Alanine	1.48 (d, J = 7.2 Hz)
GABA	1.91 (m), 2.36 (t), 2.98 (t)
Glutamine	2.12 (m), 2.46 (m), 3.64
Aspartate	2.63 (dd, J = 17.4, 9.3 Hz), 2.85 (dd, J = 17.4, 3.6 Hz)
Asparagine	2.75 (dd, J = 16.9, 8.0 Hz), 2.95 (dd, J = 16.9, 4.2 Hz)
Glycine	3.56 (s)
**Organic acids**	
Quinic acid	1.87 (dd), 1.93 (m), 2.01 (m), 3.49, 3.94, 4.09 (m)
Acetic acid	1.99 (s)
Succinic acid	2.54 (s)
Malic acid	2.57 (dd, J = 15.4, 9.4 Hz), 2.79 (dd, J = 15.4, 3.3 Hz), 4.31 (dd, J = 9.4, 3.3 Hz)
Fumaric acid	6.49 (s)
Formic acid	8.47 (s)
**Vitamins**	
Ascorbic acid (vitamin C)	4.55 (d)
**Sugars**	
Glucose	5.14 (d, J = 3.7 Hz), 4.52 (d, J = 7.9 Hz), 3.16 (dd)
Fructose	4.06 (m), 4.01 (m), 3.88 (m)
Sucrose	5.40 (d, J = 3.7 Hz), 4.13 (d, J = 7.9 Hz)
Xylose	5.08 (d, J = 3.7 Hz), 4.45 (d, J = 7.9 Hz), 3.15 (dd)
**Phenolic acids**	
Gallic acid	7.07 (s)
Syringic acid	7.29 (s)
**Flavonoids**	
Kaempferol-3-*O*-rhamnoside	7.77 (d, J = 9.4 Hz), 6.99 (d, J = 9.4 Hz), 6.45 (d, J = 1.9 Hz), 6.27 (d, J = 1.9 Hz), 5.52 (1H, d, J = 1.4 Hz, H-1″), 4.20 (1H, d, J = 1.4 Hz, H-2″), 3.70 (1H, dd, J = 3.7, 8.7 Hz, H-3″), 3.30 (2H, m, H-4″ and H-5″), and 0.90 (3H, d, J = 6.0 Hz, Me-6″).
Kaempferol analogue 1	8.02 (d, J = 8.9 Hz), 6.94 (d, J = 8.9 Hz) 6.45 (bs), 6.27 (bs)
Kaempferol analogue 2	8.05 (d, J = 8.9 Hz), 6.96 (d, J = 8.9 Hz) 6.45 (bs), 6.27 (bs)
Kaempferol analogue 3	8.18 (d, J = 8.8 Hz), 7.25 (d, J = 8.8 Hz)
Quercitrin (Quercetin-3-*O*-rhamnoside)	7.36 (d, J = 1.9 Hz), 7.31 (dd), 6.97 (d, J = 8.2 Hz), 6.36 (1H, d, J = 1.8 Hz, H-8), 6.20 (1H, d, J = 1.8 Hz, H-6), 5.30 (1H, d, J = 1.4 Hz, H-1″), 4.20 (1H, dd, J = 3.2, 1.4 Hz, H-2″), 3.70 (1H, dd, J = 9.1, 3.7 Hz, H-3″), 3.40 (1H, dd, J = 5.9, 9.6 Hz, H-5″), 3.30 (1H, dd, J = 5.9, 9.6 Hz, H-4″), 0.90 (3H, d, J = 6.0 Hz, Me-6″).
Quercetin analogue 1	7.69 (d), 7.57 (dd), 6.93, 6.25 (bs), 6.47 (bs)
Quercetin analogue 2	7.78 (d), 7.58 (dd), 6.93, 6.25 (bs), 6.47 (bs)
	7.54 (d), 7.49 (dd), 6.96 (d)
Quercetin analogue 3	7.76, 7.72 (dd), 7.30, 6.24 (d), 6.47 (d)
Epicatechin	6.17 (d), 6.14 (d)
Catechin	5.98 (d), 5.89 (d)
**Others**	
Sterols	0.68 (s)
Fatty acids	0.88, 0.96 (t), 1.79-1.35 (m), 1.59 (m), 2.06 (m), 2.18 (t), 2.82 (m, from PUFA), 5.40 (m, from UFA)
Choline	3.21 (s)
Phosphocholine	3.23 (s)
Benzene	7.21 (s) (13C = 128.1 ppm)

## Data Availability

The data presented in this study are available on request from the corresponding authors. The data are not publicly available yet because funded grants are still ongoing.

## Supplementary Materials

The following supporting information can be downloaded at: https://www.mdpi.com/article/10.3390/ijms27020907/s1.

## Abbreviations

The following abbreviations are used in this manuscript:

AD	Alzheimer’s Disease
ATCC	American Type Culture Collection
CQ	Chloroquine
DMEM	Dulbecco’s Modified Eagle’s Medium
FBS	Foetal Bovine Serum
MTT	3-(4,5-dimethylthiazol-2-yl)-2,5-diphenyltetrazolium bromide
NaN3	Sodium azide
NMR	Nuclear Magnetic Resonance
PBS	Phosphate-Buffered Saline
ROS	Reactive Oxygen Species
RPMI-1640	Roswell Park Memorial Institute Medium 1640
TNBC	Triple-Negative Breast Cancer
UPLC MS/MS	Ultra Performance Liquid Chromatography coupled to Tandem Mass Spectrometry

## References

[1] Key T.J., Bradbury K.E., Perez-Cornago A., Sinha R., Tsilidis K.K., Tsugane S. (2020). Diet, Nutrition, and Cancer Risk: What Do We Know and What Is the Way Forward?. BMJ.

[2] Giampieri F., Forbes-Hernandez T.Y., Gasparrini M., Alvarez-Suarez J.M., Afrin S., Bompadre S., Quiles J.L., Mezzetti B., Battino M. (2015). Strawberry as a Health Promoter: An Evidence Based Review. Food Funct..

[3] Rivas-García L., Navarro-Hortal M.D., Romero-Márquez J.M., Forbes-Hernández T.Y., Varela-López A., Llopis J., Sánchez-González C., Quiles J.L. (2021). Edible Flowers as a Health Promoter: An Evidence-Based Review. Trends Food Sci. Technol..

[4] Rivas-García L., Quiles J.L., Roma-Rodrigues C., Raposo L.R., Navarro-Hortal M.D., Romero-Márquez J.M., Esteban-Muñoz A., Varela-López A., García L.C., Cianciosi D. (2021). *Rosa x hybrida* Extracts with Dual Actions: Antiproliferative Effects against Tumour Cells and Inhibitor of Alzheimer Disease. Food Chem. Toxicol..

[5] Rivas-García L., Romero-Márquez J.M., Navarro-Hortal M.D., Esteban-Muñoz A., Giampieri F., Sumalla-Cano S., Battino M., Quiles J.L., Llopis J., Sánchez-González C. (2022). Unravelling Potential Biomedical Applications of the Edible Flower *Tulbaghia violacea*. Food Chem..

[6] Grosso G., Godos J., Currenti W., Micek A., Falzone L., Libra M., Giampieri F., Forbes-Hernández T.Y., Quiles J.L., Battino M. (2022). The Effect of Dietary Polyphenols on Vascular Health and Hypertension: Current Evidence and Mechanisms of Action. Nutrients.

[7] Wang L., Wei T., Zheng L., Jiang F., Ma W., Lu M., Wu X., An H. (2023). Recent Advances on Main Active Ingredients, Pharmacological Activities of *Rosa roxburghii* and Its Development and Utilization. Foods.

[8] Huang D., Li C., Chen Q., Xie X., Fu X., Chen C., Huang Q., Huang Z., Dong H. (2022). Identification of Polyphenols from *Rosa roxburghii* Tratt Pomace and Evaluation of In Vitro and In Vivo Antioxidant Activity. Food Chem..

[9] Sabahi Z., Hasan S.M.F., Ayatollahi S.A., Farmani F., Afsari A., Moein M. (2022). Improvement of Phenolic Compound Extrac-tion by Using Ion Exchange Chromatography and Evaluation of Biological Activities of Polyphenol-Enriched Fraction of *Rosa canina* Fruits. Iran J. Pharm. Res..

[10] Wang L., Li Y., Xia R., Zheng X., Li X., Wu S., Zhang Q., Li S., Deng Y., Yao Y. (2022). Component Analysis and Anti-Pulmonary Fibrosis Effects of *Rosa sterilis* Juice. Food Funct..

[11] Kaneda H., Hori M., Shinomiya H., Nakajima A., Yamazaki S., Sasaki N., Sato T., Kaneda T. (2022). *Rosa centifolia* Petal Extract Induces Endothelium-dependent and Endothelium-independent Vasorelaxation in Rat Aorta and Prevents Accumulation of Inflammatory Factors in Human Umbilical Vein Endothelial Cells. J. Food Biochem..

[12] Olech M., Pietrzak W., Nowak R. (2020). Characterization of Free and Bound Phenolic Acids and Flavonoid Aglycones in *Rosa rugosa* Thunb. Leaves and Achenes Using LC–ESI–MS/MS–MRM Methods. Molecules.

[13] Koczka N., Stefanovits-Bányai É., Ombódi A. (2018). Total Polyphenol Content and Antioxidant Capacity of Rosehips of Some *Rosa* Species. Medicines.

[14] Faur C.-A., Zăhan M., Bunea C.I., Hârșan E., Bora F.-D., Bunea A. (2024). Antiproliferative and Biochemical Evaluation of Rose Extracts: Impact on Tumor and Normal Skin Cells. Front. Plant Sci..

[15] Zamiri-Akhlaghi A., Rakhshandeh H., Tayarani-Najaran Z., Mousavi S.H. (2011). Study of Cytotoxic Properties of *Rosa damascena* Extract in Human Cervix Carcinoma Cell Line. Avicenna J. Phytomedicine.

[16] Giovannelli P., Di Donato M., Galasso G., Di Zazzo E., Bilancio A., Migliaccio A. (2018). The Androgen Receptor in Breast Cancer. Front. Endocrinol..

[17] Krishna T.G., Mahboub M.A.A. (2024). Improving Breast Cancer Diagnosis with AI Mammogram Image Analysis. Medinformatics.

[18] Noblejas-López M.D.M., Tébar-García D., López-Rosa R., Alcaraz-Sanabria A., Cristóbal-Cueto P., Pinedo-Serrano A., Rivas-García L., Galán-Moya E.M. (2023). TACkling Cancer by Targeting Selective Protein Degradation. Pharmaceutics.

[19] Butkevičiūtė A., Urbštaitė R., Liaudanskas M., Obelevičius K., Janulis V. (2022). Phenolic Content and Antioxidant Activity in Fruit of the Genus *Rosa* L. Antioxidants.

[20] Fernandes L., Casal S., Pereira J.A., Saraiva J.A., Ramalhosa E. (2020). An Overview on the Market of Edible Flowers. Food Rev. Int..

[21] Emwas A.-H.M., Bjerrum J.T. (2015). The Strengths and Weaknesses of NMR Spectroscopy and Mass Spectrometry with Particular Focus on Metabolomics Research. Metabonomics.

[22] Zhang J., Xiao Y., Guan Y., Rui X., Zhang Y., Dong M., Ma W. (2019). An Aqueous Polyphenol Extract from *Rosa rugosa* Tea Has Antiaging Effects on *Caenorhabditis elegans*. J. Food Biochem..

[23] Rivas-García L., Crespo-Antolín L., Forbes-Hernández T.Y., Romero-Márquez J.M., Navarro-Hortal M.D., Arredondo M., Llopis J., Quiles J.L., Sánchez-González C. (2023). Bioactive Properties of Tagetes Erecta Edible Flowers: Polyphenol and Antioxidant Characterization and Therapeutic Activity against Ovarian Tumoral Cells and Caenorhabditis Elegans Tauopathy. Int. J. Mol. Sci..

[24] Mazzara E., Caprioli G., Simonelli G., Mustafa A.M., Maggi F., Cespi M. (2023). Microwave Hydrodiffusion and Gravity Extraction of Vitamin C and Antioxidant Compounds from Rosehips (*Rosa canina* L.). Foods.

[25] Putnik P., Lorenzo J., Barba F., Roohinejad S., Režek Jambrak A., Granato D., Montesano D., Bursać Kovačević D. (2018). Novel Food Processing and Extraction Technologies of High-Added Value Compounds from Plant Materials. Foods.

[26] Yu K., Zhou L., Sun Y., Zeng Z., Chen H., Liu J., Zou L., Liu W. (2021). Anti-Browning Effect of *Rosa roxburghii* on Apple Juice and Identification of Polyphenol Oxidase Inhibitors. Food Chem..

[27] Cao S., Liang J., Chen M., Xu C., Wang X., Qiu L., Zhao X., Hu W. (2025). Comparative Analysis of Extraction Technologies for Plant Extracts and Absolutes. Front. Chem..

[28] Al-Oqail M.M., Farshori N.N., Al-Sheddi E.S., Al-Massarani S.M., Saquib Q., Siddiqui M.A., Al-Khedhairy A.A. (2021). Oxidative Stress Mediated Cytotoxicity, Cell Cycle Arrest, and Apoptosis Induced by *Rosa damascena* in Human Cervical Cancer HeLa Cells. Oxidative Med. Cell. Longev..

[29] Kilinc K., Demir S., Turan I., Mentese A., Orem A., Sonmez M., Aliyazicioglu Y. (2020). *Rosa canina* Extract Has Antiproliferative and Proapoptotic Effects on Human Lung and Prostate Cancer Cells. Nutr. Cancer.

[30] Kim J., Sudhakaran M., Crockett E., Doseff A. (2020). Dietary Flavonoids Targeting Triple Negative Breast Cancer. Curr. Dev. Nutr..

[31] Yin L., Duan J.-J., Bian X.-W., Yu S. (2020). Triple-Negative Breast Cancer Molecular Subtyping and Treatment Progress. Breast Cancer Res..

[32] Liu H., Li Z., Huo S., Wei Q., Ge L. (2019). Induction of G0/G1 Phase Arrest and Apoptosis by CRISPR/Cas9-mediated Knockout of CDK2 in A375 Melanocytes. Mol. Clin. Oncol..

[33] Perillo B., Di Donato M., Pezone A., Di Zazzo E., Giovannelli P., Galasso G., Castoria G., Migliaccio A. (2020). ROS in Cancer Therapy: The Bright Side of the Moon. Exp. Mol. Med..

[34] Ajayi R.O., Ogunjobi T.T. (2024). Environmental Exposures and Cancer Risk: A Comprehensive Review. Medinformatics.

[35] Kim B.-W., Lee E.-R., Min H.-M., Jeong H.-S., Ahn J.-Y., Kim J.-H., Choi H.-Y., Choi H., Kim E.Y., Park S.P. (2008). Sustained ERK Activation Is Involved in the Kaempferol-Induced Apoptosis of Breast Cancer Cells and Is More Evident under 3-D Culture Condition. Cancer Biol. Ther..

[36] Singaravelan N., Tollefsbol T.O. (2025). Polyphenol-Based Prevention and Treatment of Cancer Through Epigenetic and Combinatorial Mechanisms. Nutrients.

[37] Nguyen L.T., Lee Y.-H., Sharma A.R., Park J.-B., Jagga S., Sharma G., Lee S.-S., Nam J.-S. (2017). Quercetin Induces Apoptosis and Cell Cycle Arrest in Triple-Negative Breast Cancer Cells through Modulation of Foxo3a Activity. Korean J. Physiol. Pharmacol..

[38] Tsouh Fokou P.V., Kamdem Pone B., Appiah-Oppong R., Ngouana V., Bakarnga-Via I., Ntieche Woutouoba D., Flore Donfack Donkeng V., Tchokouaha Yamthe L.R., Fekam Boyom F., Arslan Ateşşahin D. (2025). An Update on Antitumor Efficacy of Catechins: From Molecular Mechanisms to Clinical Applications. Food Sci. Nutr..

[39] Sun G., Zhang S., Xie Y., Zhang Z., Zhao W. (2016). Gallic Acid as a Selective Anticancer Agent That Induces Apoptosis in SMMC-7721 Human Hepatocellular Carcinoma Cells. Oncol. Lett..

[40] Sitthisuk P., Kwankaew N., Innajak S., Nilchad K., Poorahong W., Kongdang P., Watanapokasin R. (2025). Mechanism of Anti-Cancer in Breast Cancer Cells With HER2 Overexpression by Dietary Supplement of Five Edible Plants. Food Sci. Nutr..

[41] Yun C., Lee S. (2018). The Roles of Autophagy in Cancer. Int. J. Mol. Sci..

[42] Ferro F., Servais S., Besson P., Roger S., Dumas J.-F., Brisson L. (2020). Autophagy and Mitophagy in Cancer Metabolic Remodelling. Semin. Cell Dev. Biol..

[43] Cordani M., Donadelli M., Strippoli R., Bazhin A.V., Sánchez-Álvarez M. (2019). Interplay between ROS and Autophagy in Cancer and Aging: From Molecular Mechanisms to Novel Therapeutic Approaches. Oxidative Med. Cell. Longev..

[44] de Campos Vidal B., Mello M.L.S. (2019). Toluidine Blue Staining for Cell and Tissue Biology Applications. Acta Histochem..

