# Supplementary material for: Rosa x hybrida: A New Tool for Functional Food Development with Triple-Negative Breast Antitumoral Implications
Source: Int J Mol Sci. 2026 Jan 16;27(2):907. doi: 10.3390/ijms27020907 (PMC12841513; doi:10.3390/ijms27020907)
Supplement: Supplementary file 1 [file ijms-27-00907-s001.zip › ijms-3946827-supplementary.pdf]

Table S1. ***Rosa x hybrida* as a new tool for functional food development with triple-negative breast antitumoral implications.**

**Cell line**	**Sensible**	**Resistant**	**Doses (µg/mL)**	**Exposition time (h)**
MDA-MB-231	X		50<br>100<br>250<br>500	72,24,48
Hs578T		X	50<br>100<br>250<br>500	72
BT-549	X		25<br>50<br>100	72
MCF10A			50<br>100<br>250<br>500	72

Control

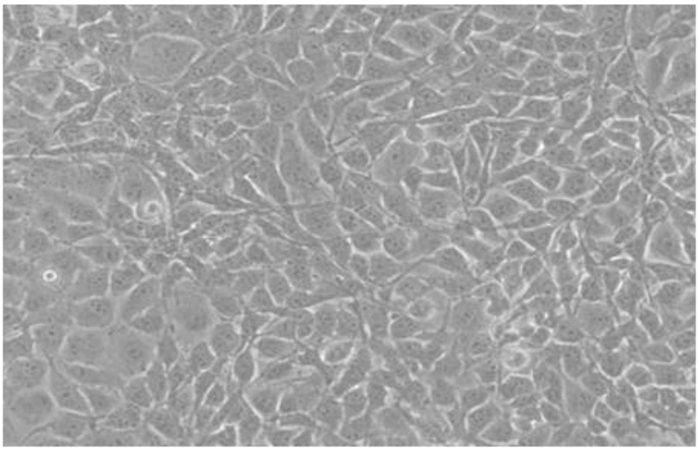

Rosa x hybrida

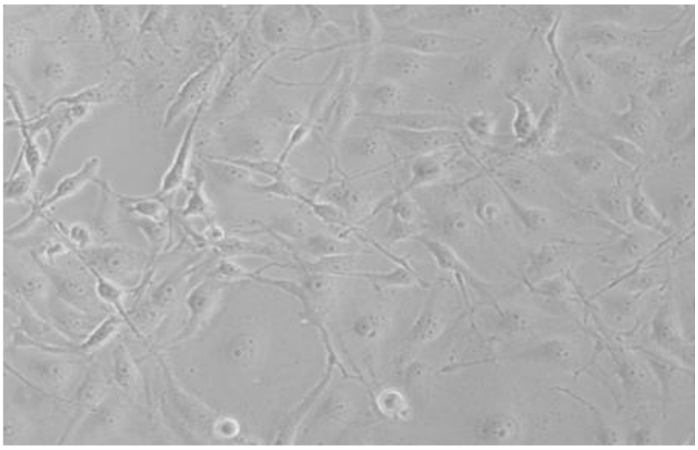

Supplementary Figure S1. Optical microscopy images of MDA-MB-231 cells treated with the IC_{50} of *Rosa x hybrida* extract.

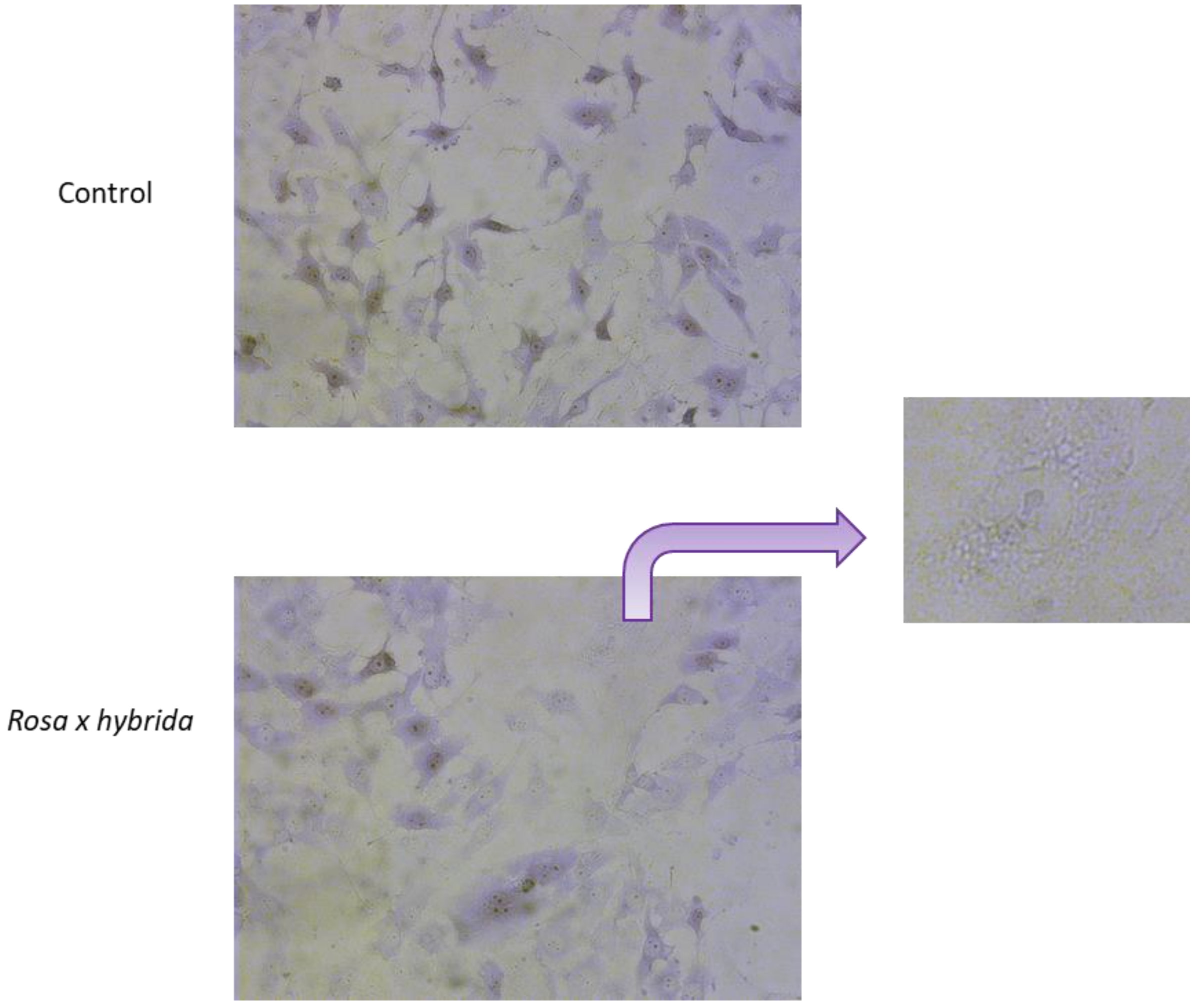

Supplemental Figure S2. Optical microscopy images of MDA-MB-231 cells treated with the IC_{50} of *Rosa x hybrida* extract stained with toluidine blue. Detailed engrossed cell.
